# Supplementary material for: Downregulation of chemokine receptor 9 facilitates CD4+CD8αα+ intraepithelial lymphocyte development
Source: Nat Commun. 2023 Aug 24;14:5152. doi: 10.1038/s41467-023-40950-2 (PMC10449822; doi:10.1038/s41467-023-40950-2)
Supplement: Supplementary file 1 — Supplementary Information [file 41467_2023_40950_MOESM1_ESM.pdf]

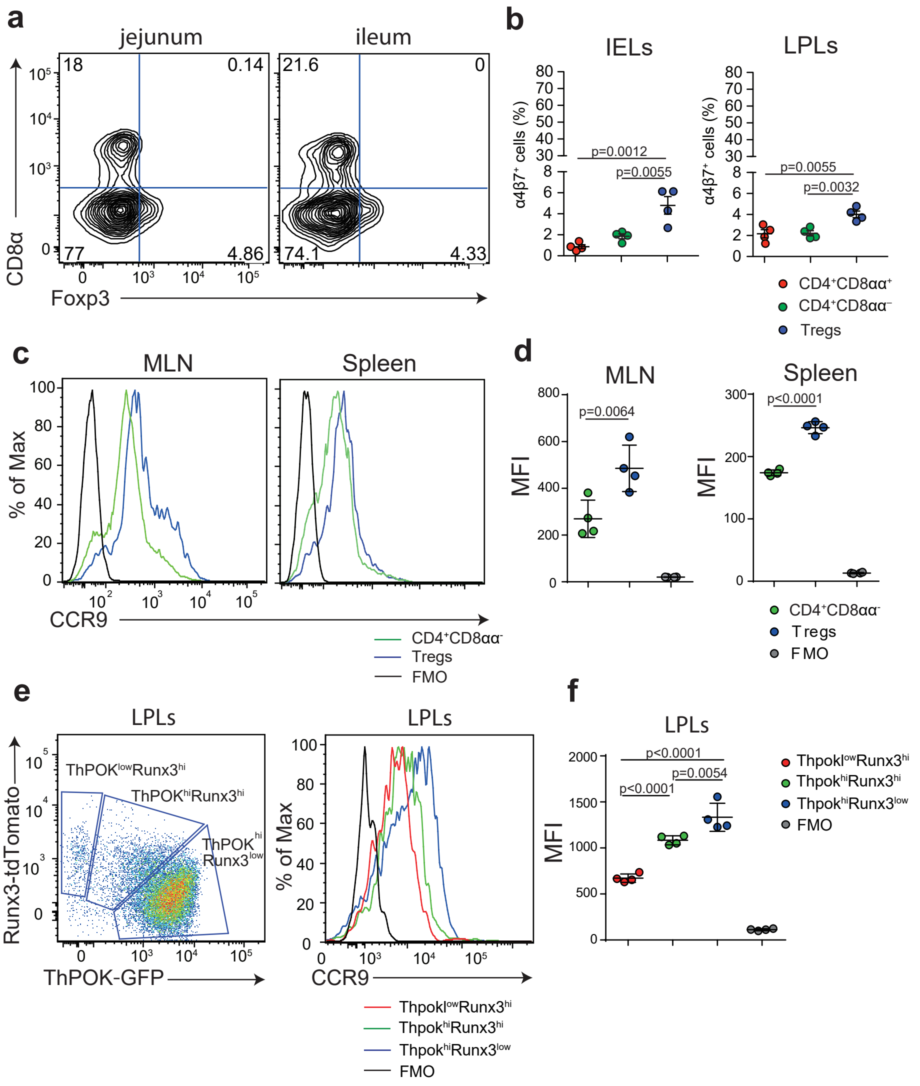

Supplementary Fig. 1. CCR9 is differentially expressed in the CD4⁺ IEL population (**a**) Contour plots show surface CD8α and intracellular Foxp3 expression of TCRβ^{+}CD4^{+}CD8β^{-} intraepithelial lymphocytes (IELs) of the jejunum and ileum. CD4^{+}CD8αα^{+}, CD4^{+}CD8αα^{-}, and Treg populations were analyzed for CCR9 and α4β7 expression. (**b**) Frequency of α4β7^{+} cells among CD4^{+}CD8αα^{+}, CD4^{+}CD8αα^{-}, and Tregs of IELs and lamina propria lymphocytes (LPLs) (*n*=4 C57BL/6J mice for IELs and LPLs analysis, 10 weeks old). Data are presented as mean ± SEM. (**c**) Histograms show the CCR9 expression among CD4^{+}CD8αα^{-} T cells (green line), and Tregs (blue line) of the MLN and spleen. FMO control is shown as black line. (**d**) Graphs show the mean fluorescence intensity (MFI) of CCR9 among CD4^{+}CD8αα^{-} T cells, Tregs, and FMO control of MLN and spleen (*n*=4 C57BL/6J mice for MLN and splenocytes analysis, 10 weeks old). Data are presented as mean ± SD. (**e**) Left; pseudocolor plot shows three subsets (ThPOK^{low}Runx3^{hi}, ThPOK^{hi}Runx3^{hi}, and ThPOK^{hi}Runx3^{low}) of CD4^{+} SI LPLs according to the expression of ThPOK and Runx3. Right; histogram shows the expression of CCR9 among ThPOK^{low}Runx3^{hi} (red line), ThPOK^{hi}Runx3^{hi} (green line), and ThPOK^{hi}Runx3^{low} (blue line) subsets of CD4^{+} SI LPLs. FMO control is shown as black line. (**f**) Graph shows the MFI of CCR9 among ThPOK^{low}Runx3^{hi}, ThPOK^{hi}Runx3^{hi}, ThPOK^{hi}Runx3^{low}, and FMO control of SI LPLs (*n*=4 *Thpok*^{GFP}:*Runx3*^{tdTomato} reporter mice, 10 weeks old). Data are presented as mean ± SD. One-way ANOVA with Tukey's multiple comparisons post-hoc test was applied. Source data are provided as a Source Data file.

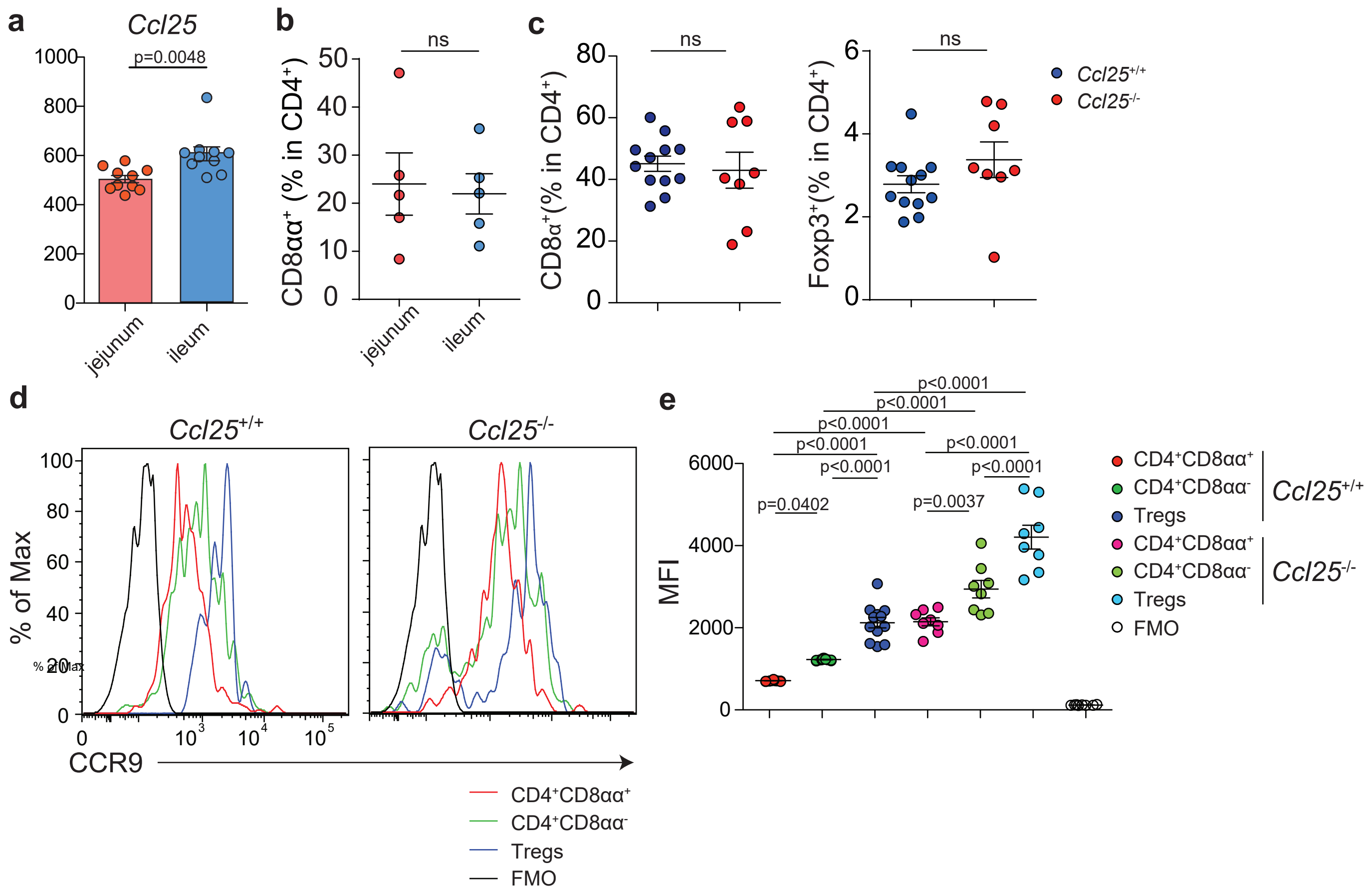

Supplementary Fig. 2. CCL25 is dispensable for CD4$^+$CD8αα$^+$ IEL differentiation. (**a**) Graph shows relative expression of *Ccl25* in the jejunum and ileum of C57BL/6J mice (10 weeks old). Quantitative real-time PCR experiments were performed in duplicate in each sample, and each dot represents the mean of the duplicate (*n*=10). (**b**) Frequency of CD8α$^+$ population among TCRβ$^+$CD4$^+$CD8β$^-$ IELs in the jejunum and ileum (*n*=5 C57BL/6J mice, 10 weeks old). (**c**) Frequency of CD8α$^+$ or Foxp3$^+$ populations among TCRβ$^+$CD4$^+$CD8β$^-$ SI IELs of *Ccl25*$^{+/+}$ and *Ccl25*$^{-/-}$ mice (*n*=12 mice for *Ccl25*$^{+/+}$ group, n=8 mice for *Ccl25*$^{-/-}$ group, 10 weeks old). (**d**) Histograms show the CCR9 level among TCRβ$^+$CD4$^+$CD8α$^+$CD8β$^-$Foxp3$^-$ (CD4$^+$CD8αα$^+$; red line), TCRβ$^+$CD4$^+$CD8α$^-$CD8β$^-$Foxp3$^-$ (CD4$^+$CD8αα$^-$; green line), and TCRβ$^+$CD4$^+$CD8α$^-$CD8β$^-$ Foxp3$^+$ (Tregs ; blue line) of IELs in *Ccl25*$^{+/+}$ and *Ccl25*$^{-/-}$ mice. Fluorescence minus one (FMO) control is shown as black line. (**e**) Graph shows the MFI of CCR9 among CD4$^+$CD8αα$^+$, CD4$^+$CD8αα$^-$, Tregs, and FMO control of IELs in *Ccl25*$^{+/+}$ and *Ccl25*$^{-/-}$ mice (*n*=12 mice for *Ccl25*$^{+/+}$ group, n=8 mice for *Ccl25*$^{-/-}$ group, 10 weeks old). Data are presented as mean ± SEM. The two-sided Student's t-test (**b, c**), or one-way ANOVA with Tukey's multiple comparisons post-hoc test (**e**) was applied. Source data are provided as a Source Data file.

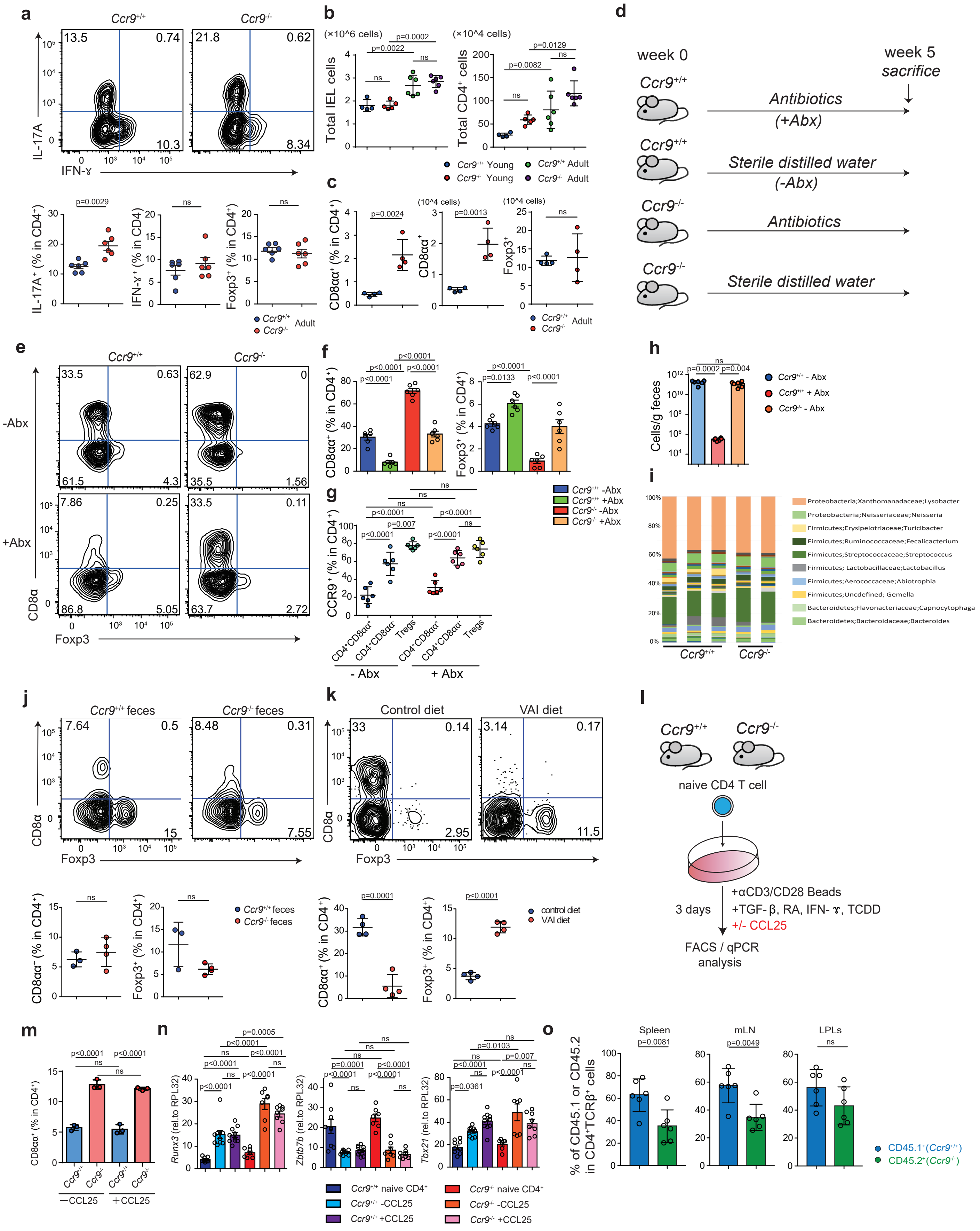

a
Ccr9+/+
Ccr9-/-
13.5
0.74
10.3
21.8
0.62
8.34
IL-17A
IFN-γ
p=0.0029
ns
ns
IL-17A+ (% in CD4+)
IFN-γ+ (% in CD4)
Foxp3+ (% in CD4+)
Ccr9+/+
Ccr9-/-
Adult
b
(×10^6 cells)
(×10^4 cells)
p=0.0002
p=0.0022
ns
ns
p=0.0129
ns
p=0.0082
ns
Total IEL cells
Total CD4+ cells
Ccr9+/+ Young
Ccr9-/- Young
Ccr9+/+ Adult
Ccr9-/- Adult
c
(10^4 cells)
(10^4 cells)
p=0.0024
p=0.0013
ns
CD8αα+ (% in CD4+)
CD8αα+
Foxp3+
Ccr9+/+
Ccr9-/-
Adult
d
week 0
week 5
sacrifice
Ccr9+/+
Antibiotics
(+Abx)
Ccr9+/+
Sterile distilled water
(-Abx)
Ccr9-/-
Antibiotics
Ccr9-/-
Sterile distilled water
e
Ccr9+/+
Ccr9-/-
-Abx
+Abx
33.5
0.63
61.5
4.3
62.9
0
35.5
1.56
7.86
0.25
86.8
5.05
33.5
0.11
63.7
2.72
CD8α
Foxp3
f
p<0.0001
p<0.0001
p<0.0001
p<0.0001
p<0.0001
p=0.0133
p<0.0001
CD8αα+ (% in CD4+)
Foxp3+ (% in CD4+)
Ccr9+/+ -Abx
Ccr9+/+ +Abx
Ccr9-/- -Abx
Ccr9-/- +Abx
g
ns
ns
ns
p<0.0001
p<0.0001
p=0.007
ns
p<0.0001
p<0.0001
CCR9+ (% in CD4+)
CD4+CD8αα+
CD4+CD8αα-
Tregs
- Abx
+ Abx
h
ns
p=0.0002
p=0.004
Cells/g feces
Ccr9+/+ - Abx
Ccr9+/+ + Abx
Ccr9-/- - Abx
i
Proteobacteria;Xanthomanadaceae;Lysobacter
Proteobacteria;Neisseriaceae;Neisseria
Firmicutes;Erysipelotriaceae;Turicibacter
Firmicutes;Ruminococcaceae;Fecalicacterium
Firmicutes;Streptococcaceae;Streptococcus
Firmicutes; Lactobacillaceae;Lactobacillus
Firmicutes;Aerococcaceae;Abiotrophia
Firmicutes;Uncdefined; Gemella
Bacteroidetes;Flavonacteriaceae;Capnocytophaga
Bacteroidetes;Bacteroidaceae;Bacteroides
Ccr9+/+
Ccr9-/-
j
Ccr9+/+ feces
Ccr9-/- feces
7.64
0.5
15
8.48
0.31
7.55
CD8α
Foxp3
ns
ns
CD8αα+ (% in CD4+)
Foxp3+ (% in CD4+)
Ccr9+/+ feces
Ccr9-/- feces
k
Control diet
VAI diet
33
0.14
2.95
3.14
0.17
11.5
CD8α
Foxp3
p=0.0001
p<0.0001
CD8αα+ (% in CD4+)
Foxp3+ (% in CD4+)
control diet
VAI diet
l
Ccr9+/+
Ccr9-/-
naive CD4 T cell
3 days
+αCD3/CD28 Beads
+TGF-β, RA, IFN-γ, TCDD
+/- CCL25
FACS / qPCR
analysis
m
p<0.0001
p<0.0001
ns
ns
CD8αα+ (% in CD4+)
Ccr9+/+
Ccr9-/-
Ccr9+/+
Ccr9-/-
−CCL25
+CCL25
n
Runx3 (rel.to RPL32)
Zbtb7b (rel.to RPL32)
Tbx21 (rel.to RPL32)
Ccr9+/+ naive CD4+
Ccr9+/+ -CCL25
Ccr9+/+ +CCL25
Ccr9-/- naive CD4+
Ccr9-/- -CCL25
Ccr9-/- +CCL25
o
Spleen
p=0.0081
mLN
p=0.0049
LPLs
ns
% of CD45.1 or CD45.2 in CD4+TCRβ+ cells
CD45.1+(Ccr9+/+)
CD45.2+(Ccr9-/-)

Supplementary Fig. 3. CD4$^+$CD8αα$^+$ IELs were induced in an RA- and microbiome-dependent manner in *Ccr9$^{-/-}$* mice. (**a**) Contour plots show intracellular IL-17A and IFN-ϒ expression in CD45$^+$TCRβ$^+$CD4$^+$ in SI lamina propria cells (LPLs) from *Ccr9$^{+/+}$* and *Ccr9$^{-/-}$* adult mice. Frequency of IL-17A$^+$ or IFN-γ$^+$ subsets among CD45$^+$TCRβ$^+$CD4$^+$ cells and Foxp3$^+$ subsets among TCRβ$^+$CD4$^+$CD8β$^-$ SI LPLs (*n*=6 mice for each group, 10 weeks old). Data are presented as mean ± SEM. (**b**) Abundance of SI IELs and CD4$^+$ SI IEL cells in *Ccr9$^{+/+}$* and *Ccr9$^{-/-}$* mice (Young, analyzed at 7 weeks old; Adult, analyzed at 10 weeks old. *n*=4 mice for young *Ccr9$^{+/+}$* group, *n*=5 mice for young *Ccr9$^{-/-}$* group, *n*=6 mice for adult mice group). Data are presented as mean ± SD. (**c**) Frequency and abundance of CD4$^+$CD8αα$^+$ SI LPLs and abundance of Foxp3$^+$ SI LPLs in *Ccr9$^{+/+}$* and *Ccr9$^{-/-}$* adult mice (*n*=4 mice for each group, 10weeks old). Data are presented as mean ± SD. (**d**-**h**) *Ccr9$^{+/+}$* and *Ccr9$^{-/-}$* mice were fed with water (-Abx) or antibiotics (+Abx) from 7 weeks old to 12 weeks old and analyzed at 12 weeks old. (**d**) Scheme of experimental design. (**e**) Contour plots show surface CD8α and intracellular Foxp3 expression in TCRβ$^+$CD4$^+$CD8β$^-$ SI IELs of *Ccr9$^{+/+}$* and *Ccr9$^{-/-}$* mice (Top, -Abx group; bottom, +Abx group). (**f**) Frequency of CD8α or Foxp3 subsets among TCRβ$^+$CD4$^+$CD8β$^-$ SI IELs from the –Abx and +Abx groups of *Ccr9$^{+/+}$* and *Ccr9$^{-/-}$* mice (*n*=6 mice for each group). Data are presented as mean ± SEM. (**g**) Frequency of the CCR9$^+$ subset among Foxp3$^-$TCRβ$^+$CD4$^+$CD8α$^+$CD8β$^-$ (CD4$^+$CD8αα$^+$), Foxp3$^-$TCRβ$^+$CD4$^+$CD8α$^-$CD8β$^-$ (CD4$^+$CD8αα$^-$), and Foxp3$^+$TCRβ$^+$CD4$^+$CD8α$^-$CD8β$^-$ (Tregs) SI IELs from the –Abx and +Abx groups of *Ccr9$^{+/+}$* and *Ccr9$^{-/-}$* mice (*n*=6 mice for each group). Data are presented as mean ± SD. (**h**) Bacterial load of feces from –Abx and +Abx *Ccr9$^{+/+}$* mice and –Abx *Ccr9$^{-/-}$* mice, as determined by qPCR at 12 weeks (*n*=6 mice for each group). Data are presented as mean ± SEM. (**i**) Feces obtained from *Ccr9$^{+/+}$* and *Ccr9$^{-/-}$* mice were analyzed by 16S rRNA gene sequencing (*n*=3 biologically independent samples obtained from *Ccr9$^{+/+}$* mice, and *n*=2 biologically independent samples obtained from *Ccr9$^{-/-}$* mice). (**j**) Feces obtained from *Ccr9$^{+/+}$* and *Ccr9$^{-/-}$* mice were transferred to germ-free mice and analyzed three weeks after the fecal transfer. Representative contour plots show surface CD8α and intracellular Foxp3 expression in TCRβ$^+$CD4$^+$CD8β$^-$ SI IELs (top). Frequency of CD8α$^+$ or Foxp3$^+$ subsets among TCRβ$^+$CD4$^+$CD8β$^-$ SI IELs from *Ccr9$^{+/+}$* and *Ccr9$^{-/-}$* fecal transferred mice (bottom) (*n*=3 mice for *Ccr9$^{+/+}$* feces group, *n*=4 mice for *Ccr9$^{-/-}$* feces group). Data are presented as mean ± SD. (**k**) Representative contour plots show surface CD8α and intracellular Foxp3 expression in TCRβ$^+$CD4$^+$CD8β$^-$ SI IELs from *Ccr9$^{-/-}$* mice fed a control or VAI diet and analyzed at 8 weeks old (top). Frequency of CD8α$^+$ or Foxp3$^+$ subsets among TCRβ$^+$CD4$^+$CD8β$^-$ SI IELs from *Ccr9$^{-/-}$* mice fed a control or VAI diet (bottom) (*n*=4 mice for each group). Data are presented as mean ± SD. (**l**) Scheme of the experimental design. Naïve CD4$^+$ T cells were obtained from *Ccr9$^{+/+}$* and *Ccr9$^{-/-}$* mice and cultured with anti-CD3/CD28, transforming growth factor-β (TGF-β), retinoic acid (RA), 2,3,7,8-tetrachlododibenzodioxin (TCDD), and IFN-ϒ (IEL-differentiating conditions), with or without CCL25. (**m**) Frequency of CD8α$^+$ cells among TCRβ$^+$CD4$^+$CD8β$^-$ cultured cells from *Ccr9$^{+/+}$* and *Ccr9$^{-/-}$* mice. Three independent experiments were performed in triplicate. Each dot (*n*=3) represents the mean of triplicate experiments. Data are presented as mean ± SD. (**n**) Relative expression of *Runx3, Zbtb7b*, and *Tbx21* in naïve CD4$^+$ T cells and cultured cells from *Ccr9$^{+/+}$* and *Ccr9$^{-/-}$* mice. Quantitative real-time PCR experiments were performed in duplicate in each sample, and each dot represents the mean of the duplicate (*n*=10). Data are presented as mean ± SEM. (**o**) Cells were obtained from bone marrow of *Cd45.1$^+$ Ccr9$^{+/+}$* mice and *Cd45.2$^+$ Ccr9$^{-/-}$* mice and mixed 1:1 ratio. Mixed bone marrow cells were transferred to the lethally irradiated (11Gy) C57BL/6J host mice and mice were analyzed 4 weeks after transfer (*n*=6 mice). Graphs show the percentage of CD45.1$^+$ and CD45.2$^+$ cells among TCRβ$^+$CD4$^+$CD8β$^-$ SI LPLs, MLN cells, and splenocytes. Data are presented as mean ± SD. The two-sided Student's t test (**a, c, j, k, o**) or one-way ANOVA with Tukey's multiple comparisons post-hoc test (**b, f, g, h, m, n**) was applied. Source data are provided as a Source Data file.

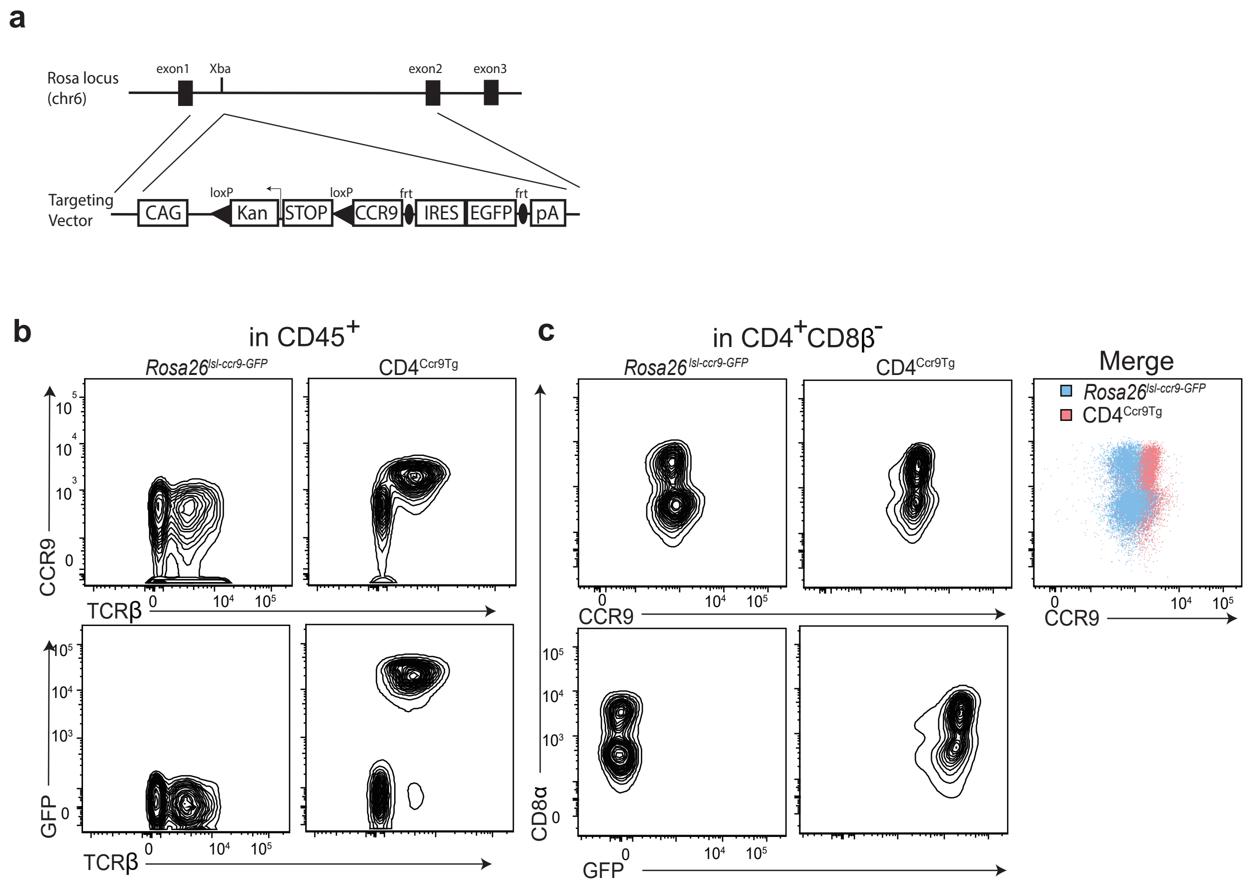

Supplementary Fig. 4 Forced CCR9 expression does not reduce CD4⁺CD8α⁺ IEL abundance. (**a**) Scheme for the strategy used to generate a $Rosa26^{lsl\text{-}Ccr9\text{-}GFP}$ mouse strain and structure of the targeting vector. CAG, CAG promoter; Kan, kanamycin resistance gene; STOP, stop cassette; IRES, internal ribosome entry site; EGFP, enhanced green fluorescent protein; pA, poly A; frt, FLP recombination target. (**b**) Surface TCRβ and CCR9 expression among $CD45^{+}$ SI IELs of $Rosa26^{lsl\text{-}Ccr9\text{-}GFP}$ and $CD4^{Ccr9Tg}$ mice (top). Surface TCRβ and GFP expression among $CD45^{+}$ SI IELs of $Rosa26^{lsl\text{-}Ccr9\text{-}GFP}$ and $CD4^{Ccr9Tg}$ mice (bottom). (**c**) Surface CD8α, CCR9, and GFP expression among $TCR\beta^{+}CD4^{+}CD8\beta^{-}$ SI IELs of $Rosa26^{lsl\text{-}Ccr9\text{-}GFP}$ and $CD4^{Ccr9Tg}$ mice. Merged plot shows surface expression of CCR9 among $TCR\beta^{+}CD4^{+}CD8\beta^{-}$ SI IELs of $Rosa26^{lsl\text{-}Ccr9\text{-}GFP}$ (blue) and $CD4^{Ccr9Tg}$ mice (red).

a

	IELs	LPLs	MLN	Spleen
Ccr9+/+_#1	4843	2256	1106	953
Ccr9+/+_#2	1856	681		
Ccr9+/+_#3	2097	931		
Ccr9-/-_#1	5377	2468	976	615
Ccr9-/-_#2	1928	582		
Ccr9-/-_#3	2048	810		

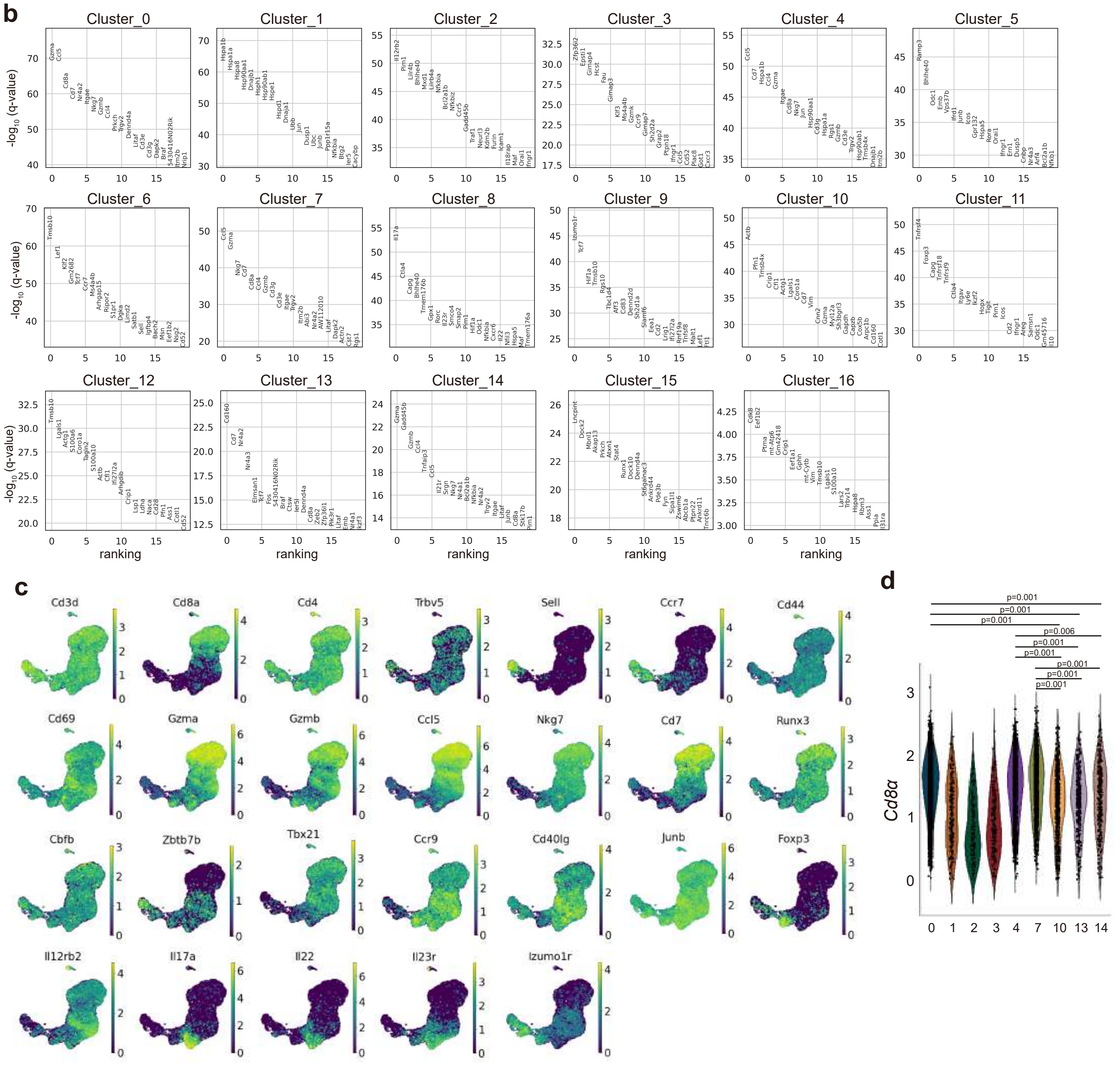

Supplementary Fig. 5. Featured gene expression of CD4⁺ T cells in *Ccr9*+/+ and *Ccr9*–/– mice. (**a**) Droplet-based scRNA-seq was performed using the Chromium 10X platform. CD4⁺ T cells from splenocytes, MLN cells, LPLs, and IELs were sorted from *Ccr9*+/+ and *Ccr9*–/– mice for scRNA-seq. Table shows the cell numbers in each sample and the strain. (**b**) Cells were sorted by gene expression similarities, and 17 clusters were identified based on top DEGs. Top 20 differential expressed genes in each cluster were listed. The y-axis displays the –log10(q-value) for each gene. (**c**) Expression of representative genes in sequenced cells is shown for cell type classification. (**d**) *Cd8a* expression of clusters 0, 4, and 7 (CD4⁺CD8αα⁺ T cells); clusters 1, 2, and 3 (CD4⁺CD8αα⁻ T cells); and clusters 10, 13, and 14 (CD4⁺CD8αα^int T cells) among IELs (*n*=3 biologically independent IEL samples for each strain). One-way ANOVA with Tukey's multiple comparisons post-hoc test (**d**) was applied. All data of exact p values (**d**) are provided in Supplementary Table 2. Source data are provided as a Source Data file.

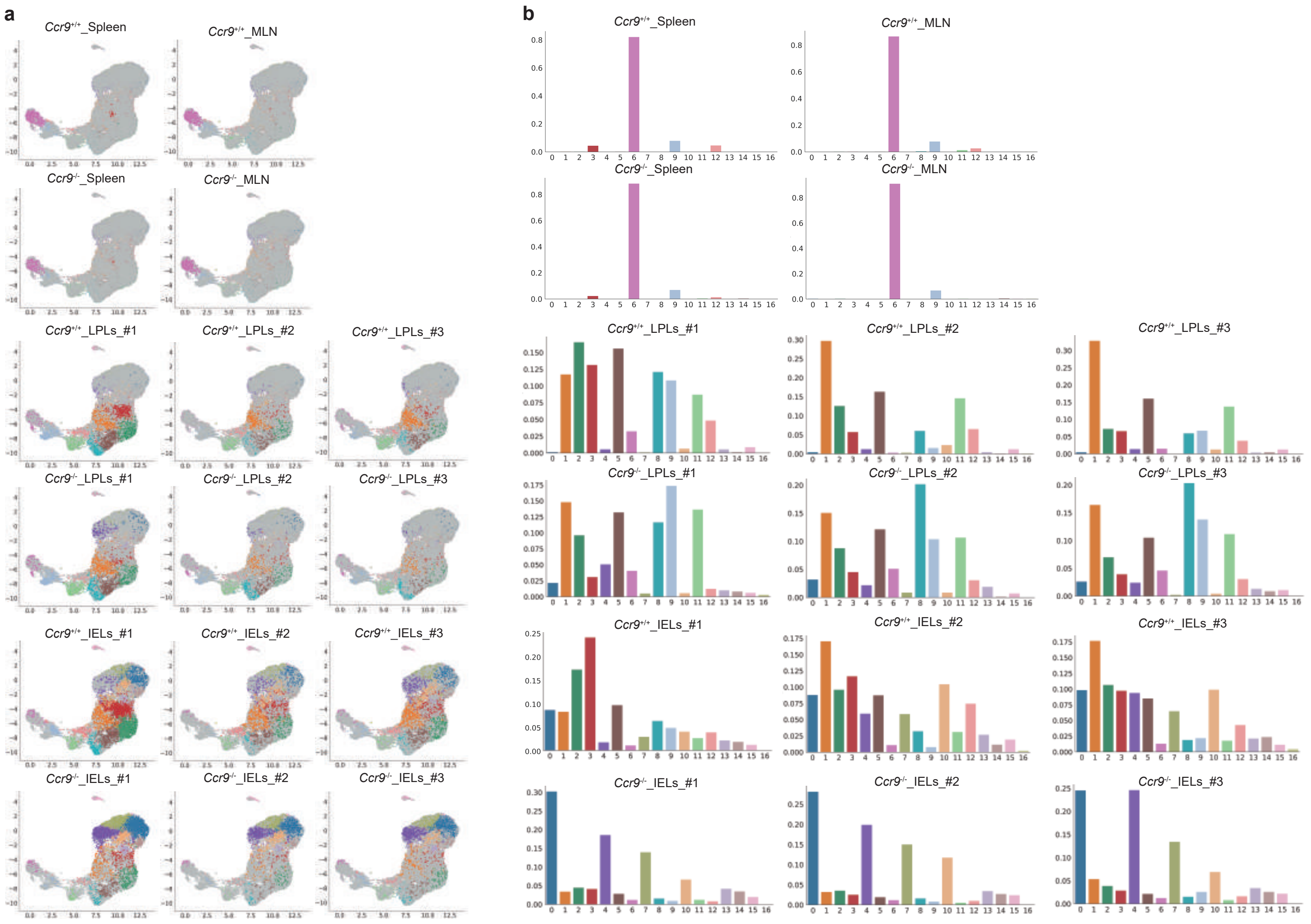

Supplementary Fig. 6. Single cell RNA-seq data of individual *Ccr9*$^{+/+}$ and *Ccr9*$^{-/-}$ mice. (**a**) UMAP of scRNA-seq data within each of the four sites from individual mice. (**b**) Proportion of each subset in (**a**). (**a, b**; *n*=3 for *Ccr9*$^{+/+}$ IELs, *n*=3 for *Ccr9*$^{+/+}$ LPLs, *n*=1 for *Ccr9*$^{+/+}$ MLN, *n*=1 for *Ccr9*$^{+/+}$ spleen, *n*=3 for *Ccr9*$^{-/-}$ IELs, *n*=3 for *Ccr9*$^{-/-}$ LPLs, *n*=1 for *Ccr9*$^{-/-}$ MLN, and *n*=1 for *Ccr9*$^{-/-}$ spleen).

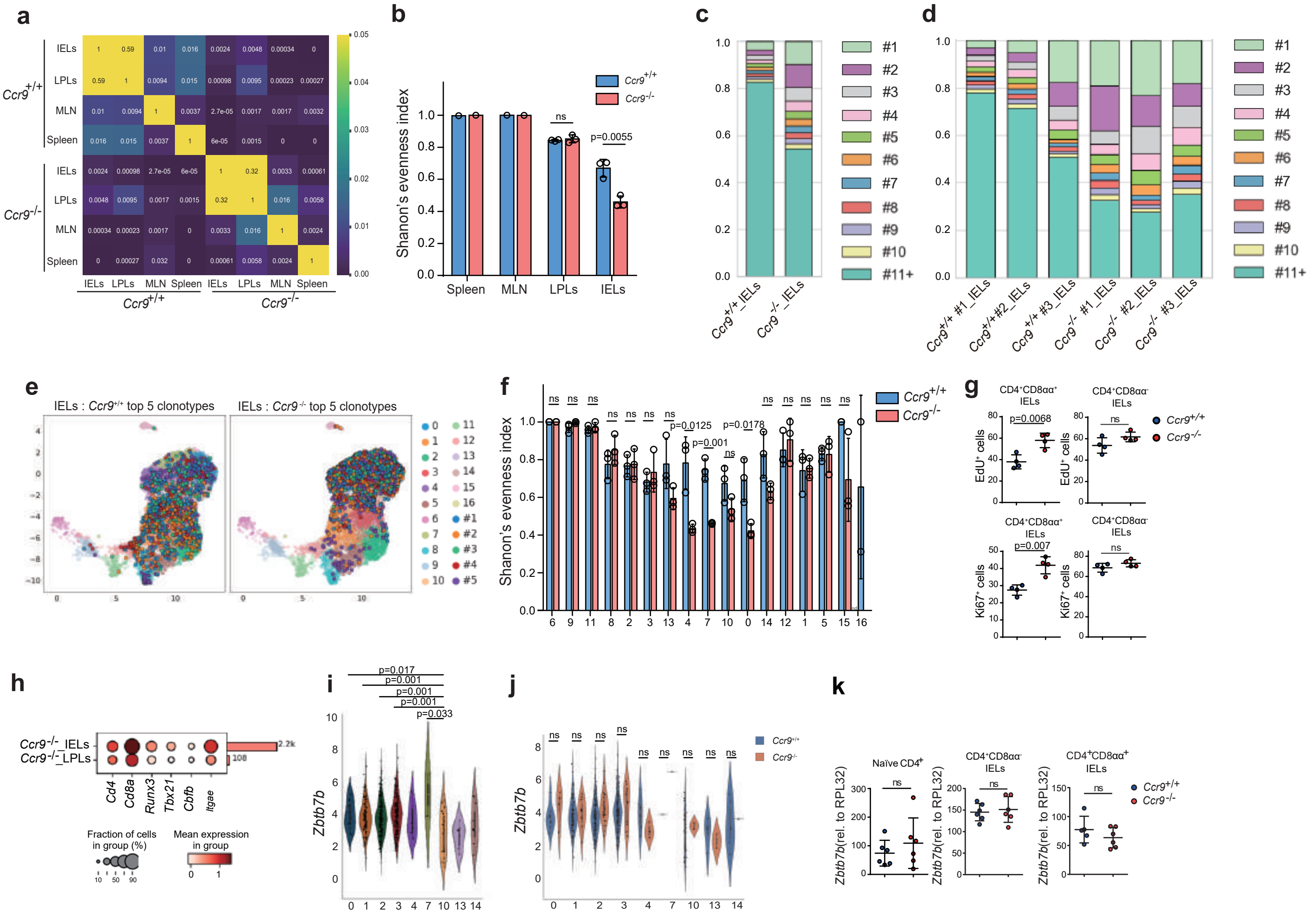

Supplementary Fig. 7. Characteristics of CD4⁺ IELs in *Ccr9*⁺/⁺ *and Ccr9*⁻/⁻ mice. (**a**) Normalized Morisita-Horn index (MHI) of paired αβTCR per population from *Ccr9*⁺/⁺ and *Ccr9*⁻/⁻ mice. (**b**) Bar graph represents TCR diversity analysis indicated by Shannon's evenness index in CD4⁺ T cells from the spleen, MLN, LPLs, and IELs of *Ccr9*⁺/⁺ and *Ccr9*⁻/⁻ mice (*n*=3 for *Ccr9*⁺/⁺ IELs, *n*=3 for *Ccr9*⁺/⁺ LPLs, *n*=1 for *Ccr9*⁺/⁺ MLN, *n*=1 for *Ccr9*⁺/⁺ spleen, *n*=3 for *Ccr9*⁻/⁻ IELs, *n*=3 for *Ccr9*⁻/⁻ LPLs, *n*=1 for *Ccr9*⁻/⁻ MLN, and *n*=1 for *Ccr9*⁻/⁻ spleen). Data are presented as mean ± SD. (**c**) Bar graph representing the ratio of each clonotype among CD4⁺ IELs summed from three animals in each strain. (**d**) Bar graph representing the ratio of each clonotype among CD4⁺ IELs of individual *Ccr9*⁺/⁺ and *Ccr9*⁻/⁻ mice (*n*=3 in each group). (**e**) The top 5 clonotypes (#1–#5) among CD4⁺ IELs are shown (left, *Ccr9*⁺/⁺ mice; right, *Ccr9*⁻/⁻ mice) on UMAP. (**f**) TCR diversity analysis indicated by Shannon's evenness index in each cluster of IELs among *Ccr9*⁺/⁺ and *Ccr9*⁻/⁻mice (*n*=3 for *Ccr9*⁺/⁺ IELs, *n*=3 for *Ccr9*⁻/⁻ IELs). Data are presented as mean ± SD. (**g**) EdU and Ki67 expression in CD4⁺CD8αα⁺ and CD4⁺CD8αα⁻ IELs from the small intestine of *Ccr9*⁺/⁺ and *Ccr9*⁻/⁻ mice after intraperitoneal injection of 400 μg EdU for 7 days. Graphs show the frequency of EdU⁺ and Ki67⁺ cells among CD4⁺CD8αα⁺ or CD4⁺CD8αα⁻ IELs from *Ccr9*⁺/⁺ and *Ccr9*⁻/⁻ mice (top, EdU expression; bottom, Ki67 expression) (*n*=4 mice for each group, 7 weeks old). Data are presented as mean ± SD. (**h**) Expression of *Cd4, Cd8a, Runx3, Tbx21, Cbfb*, and *Itgae* among *Ccr9*⁻/⁻ IELs and LPLs. (**i**) *Zbtb7b* expression in clusters 0, 4, and 7 (CD4⁺CD8αα⁺ T cells); clusters 1, 2, and 3 (CD4⁺CD8αα⁻ T cells); and clusters 10, 13, and 14 (CD4⁺CD8αα^{int} T cells) among IELs (*n*=3 for *Ccr9*⁺/⁺ IELs, *n*=3 for *Ccr9*⁻/⁻ IELs). (**j**) *Zbtb7b* expression in clusters 0, 4, and 7 (CD4⁺CD8αα⁺ T cells); clusters 1, 2, and 3 (CD4⁺CD8αα⁻ T cells); and clusters 10, 13, and 14 (CD4⁺CD8αα^{int} T cells) of *Ccr9*⁺/⁺ and *Ccr9*⁻/⁻ IELs (*n*=3 for *Ccr9*⁺/⁺ IELs, *n*=3 for *Ccr9*⁻/⁻ IELs). (**k**) Relative expression of *Zbtb7b* in splenic naïve CD4⁺ T cells, CD4⁺CD8αα⁻ and CD4⁺CD8αα⁺ SI IELs from *Ccr9*⁺/⁺ and *Ccr9*⁻/⁻ mice. Quantitative real-time PCR experiments were performed in duplicate in each sample, and each dot represents the mean of the duplicate (*n*=6). Data are presented as mean ± SD. The two-sided Student's t test (**b, f, g, j, k**) or one-way ANOVA with Tukey's multiple comparisons post-hoc test (**i**) was applied. All data of exact p values (**i**) are provided in Supplementary Table 2. Source data are provided as a Source Data file.

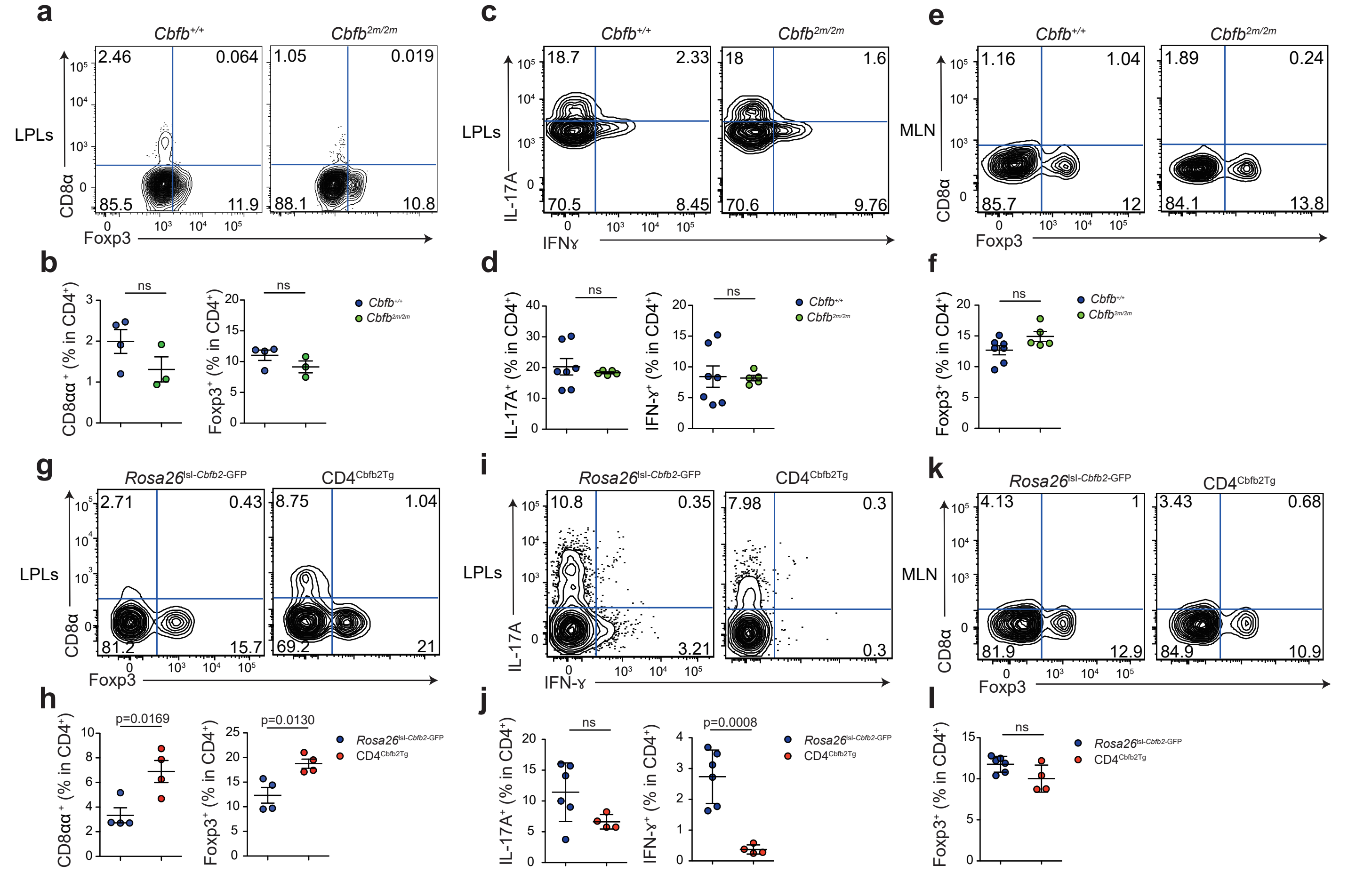

Supplementary Fig. 8. Frequency of Tregs, Th1, and Th17 cells in SI LPLs and Tregs in MLN of *Cbfb*$^{+/+}$, *Cbfb*$^{2m/2m}$, *Rosa26*$^{lsl\text{-}Cbfb2\text{-}GFP}$, and CD4^{Cbfb2Tg} mice.
(**a**) Surface CD8α and intracellular Foxp3 expression in TCRβ$^+$CD4$^+$CD8β$^-$ SI LPLs of *Cbfb*$^{+/+}$ and *Cbfb*$^{2m/2m}$ mice. (**b**) Frequency of CD8α$^+$ or Foxp3$^+$ subsets among TCRβ$^+$ CD4$^+$CD8β$^-$SI LPLs of *Cbfb*$^{+/+}$ and *Cbfb*$^{2m/2m}$ mice (*n*=4 mice for *Cbfb*$^{+/+}$ group, *n*=3 mice for *Cbfb*$^{2m/2m}$ group, 10 weeks old). Data are presented as mean ± SEM. (**c**) Intracellular IL-17A and IFN-ɣ expression in fixable viability dye (FVD)$^-$CD45$^+$TCRβ$^+$CD4$^+$ SI LPLs of *Cbfb*$^{+/+}$ and *Cbfb*$^{2m/2m}$ mice. (**d**) Graphs show the frequency of IL-17A$^+$ or IFN-γ$^+$ subsets among FVD$^-$CD45$^+$TCRβ$^+$CD4$^+$ LPLs (*n*=7 mice for *Cbfb*$^{+/+}$ group, *n*=5 mice for *Cbfb*$^{2m/2m}$ group, 10 weeks old). Data are presented as mean ± SEM. (**e**) Surface CD8α and intracellular Foxp3 expression by TCRβ$^+$CD4$^+$CD8β$^-$ cells in the MLN of *Cbfb*$^{+/+}$ and *Cbfb*$^{2m/2m}$ mice. (**f**) Foxp3$^+$ population among TCRβ$^+$CD4$^+$CD8β$^-$ cells in the MLN (*n*=7 mice for *Cbfb*$^{+/+}$ group, *n*=5 mice for *Cbfb*$^{2m/2m}$ group, 10 weeks old). Data are presented as mean ± SEM. (**g**) Surface CD8α and intracellular Foxp3 expression in TCRβ$^+$CD4$^+$ CD8β$^-$ SI LPLs of *Rosa26*$^{lsl\text{-}Cbfb2\text{-}GFP}$ and CD4^{Cbfb2Tg} mice. (**h**) Frequency of CD8α$^+$ or Foxp3$^+$ subsets among TCRβ$^+$CD4$^+$CD8β$^-$ cells in SI LPLs of *Rosa26*$^{lsl\text{-}Cbfb2\text{-}GFP}$ and CD4^{Cbfb2Tg} mice (*n*=4 mice for each group, 10 weeks old). Data are presented as mean ± SEM. (**i**) Intracellular IL-17A and IFN-ɣ expression in FVD$^-$CD45$^+$TCRβ$^+$CD4$^+$ SI LPLs from *Rosa26*$^{lsl\text{-}Cbfb2\text{-}GFP}$ and CD4^{Cbfb2Tg} mice. (**j**) Frequency of IL-17A$^+$ or IFN-γ$^+$ subsets among FVD$^-$CD45$^+$TCRβ$^+$CD4$^+$ LPLs (*n*=6 mice for *Rosa26*$^{lsl\text{-}Cbfb2\text{-}GFP}$ group, *n*=4 mice for CD4^{Cbfb2Tg} group, 10 weeks old). Data are presented as mean ± SD. (**k**) Surface CD8α and intracellular Foxp3 expression in TCRβ$^+$CD4$^+$CD8β$^-$ MLN cells from *Rosa26*$^{lsl\text{-}Cbfb2\text{-}GFP}$ and CD4^{Cbfb2Tg} mice. (**l**) Foxp3$^+$ subset among TCRβ$^+$CD4$^+$CD8β$^-$ MLN cells (*n*=6 mice for *Rosa26*$^{lsl\text{-}Cbfb2\text{-}GFP}$ group, *n*=4 mice for CD4^{Cbfb2Tg} group, 10 weeks old). Data are presented as mean ± SD. The two-sided Student's t test (**b, d, f, h, j, l**) was applied. Source data are provided as a Source Data file.

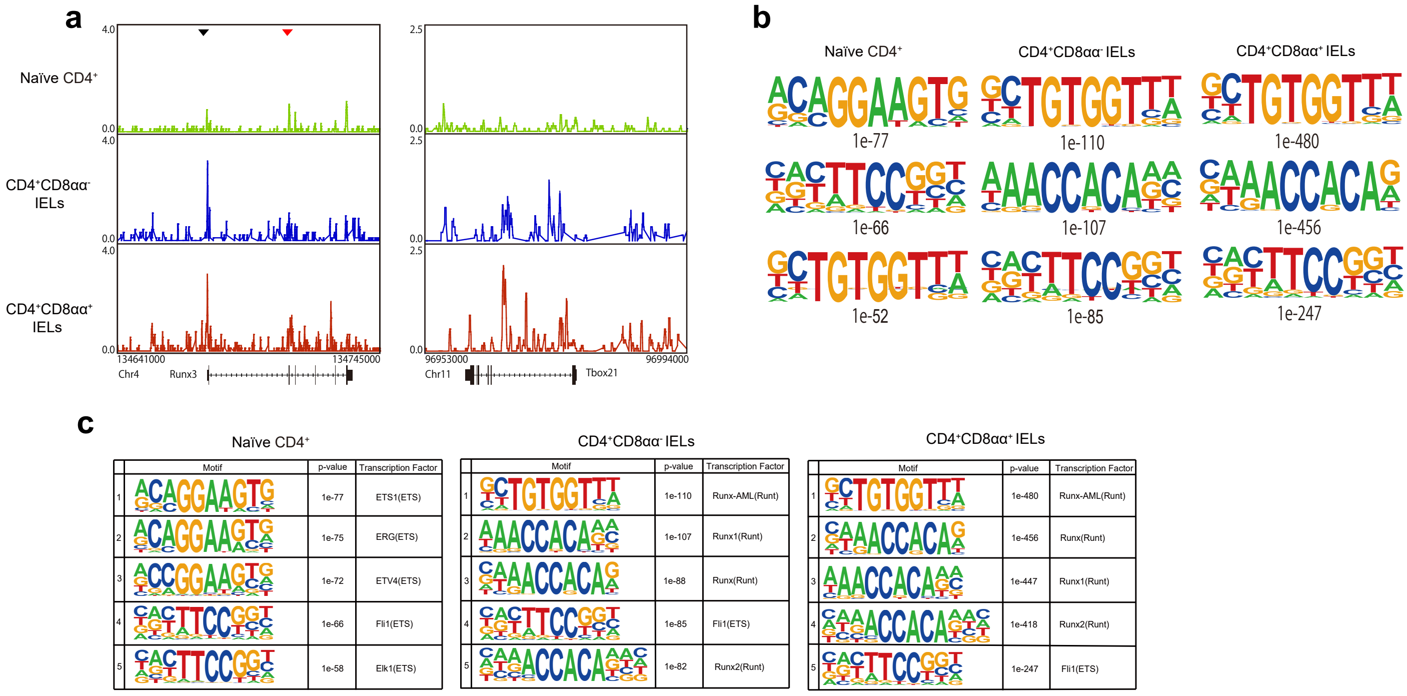

Supplementary Fig. 9. Cbfβ2 binds lesions differently during the development of CD4⁺CD8αα⁺ IELs. (**a**) Cbfβ2 chromatin immunoprecipitation sequencing (ChIP-seq) tracks for *Runx3* and *Tbx21* in splenic naïve CD4⁺ T cells, CD4⁺CD8αα⁻ IELs, and CD4⁺CD8αα⁺ IELs. Gene structure and transcriptional orientation are indicated at the bottom. The distal and proximal promoters of *Runx3* are indicated by the black and red arrowheads, respectively. (**b**) Top three unique characteristics of Cbfβ2 binding motifs in naïve CD4⁺ T cells, CD4⁺CD8αα⁻ IELs, and CD4⁺CD8αα⁺ IELs are shown with P-values. (**c**) Motif analysis of Cbfβ2 binding peaks specific to naïve CD4⁺ T cells, CD4⁺CD8αα⁻ IELs, and CD4⁺CD8αα⁺ IELs. The top five motifs are shown with P-values.

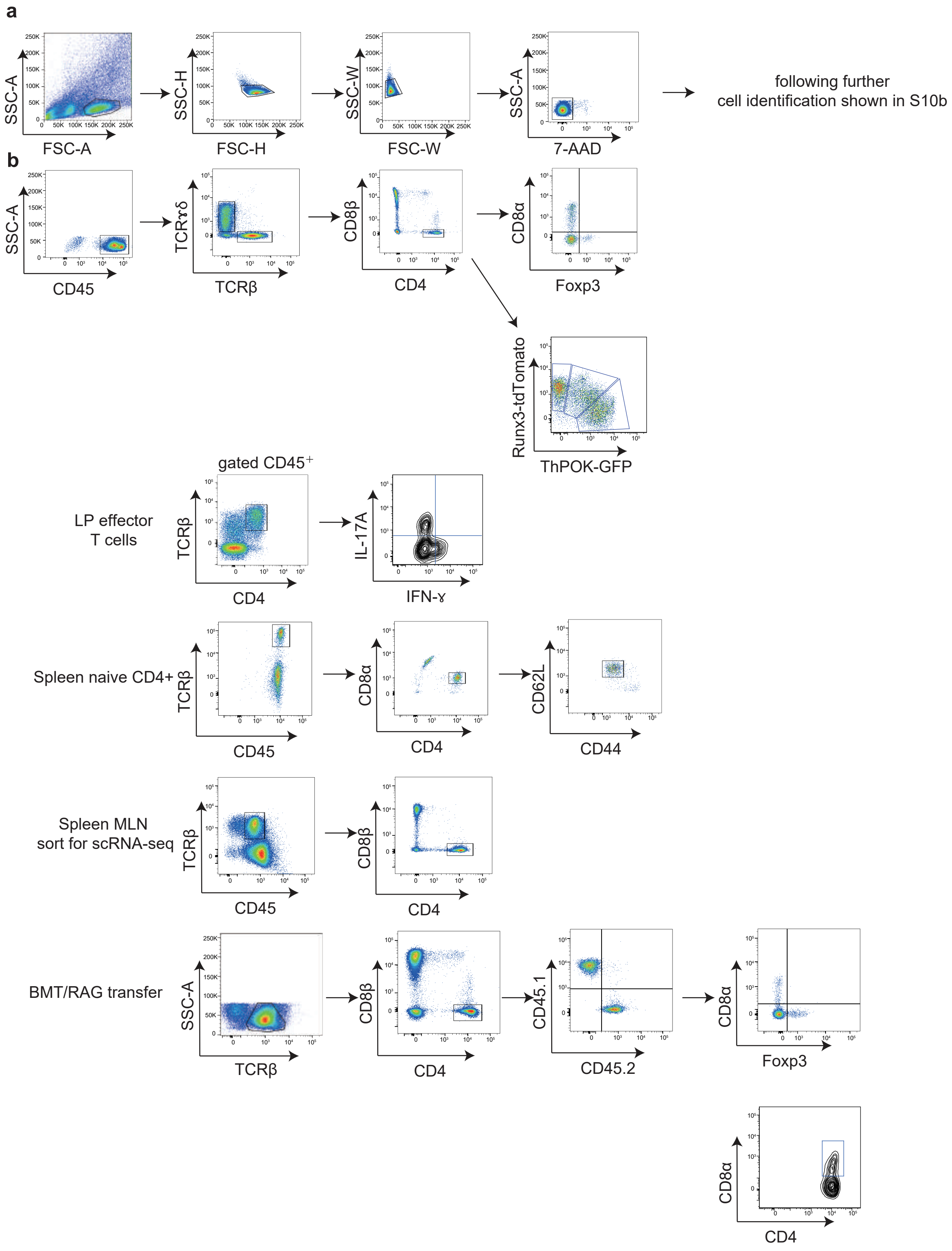

Supplementary Fig. 10 Gating strategies for cell analysis and sorting. Representative FACS plots showing the gating strategy for identifining the live cells (7-AAD or FVD negative cells) (**a**), and showing the gating strategy for analysis and sorting of the IELs, LPLs, MLN, and splenocytes (**b**).
