# Supplementary material for: Downregulation of chemokine receptor 9 facilitates CD4+CD8αα+ intraepithelial lymphocyte development
Source: Nat Commun. 2023 Aug 24;14:5152. doi: 10.1038/s41467-023-40950-2 (PMC10449822; doi:10.1038/s41467-023-40950-2)
Supplement: Supplementary file 3 — Description of Additional Supplementary Files [file 41467_2023_40950_MOESM3_ESM.pdf]

Description of Additional Supplementary Files

Supplementary Data 1. List of top significantly differentially expressed genes per cluster.

Top significantly differentially expressed genes per cluster is shown. Columns indicate cluster number, log fold change, and adjusted p value of each cluster, $P_{sig}<0.05$.

Supplementary Data 2. Statistics of gene expression of *Runx3, Tbx21*, *Cbfb, Zbtb7b,* and *CD8a* among IEL clusters.

Tables show the exact p values of *Runx3, Tbx21*, *Cbfb, Zbtb7b,* and *CD8a* among IEL clusters from clusters 0, 4, and 7 ($CD4^+CD8\alpha\alpha^+$ T cells); clusters 1, 2 and 3 ($CD4^+CD8\alpha\alpha^-$ T cells); and clusters 10, 13 and 14 ($CD4^+CD8\alpha\alpha^{int}$ T cells) as determined by one-way ANOVA with Tukey's multiple comparisons post-hoc test.

Supplementary Data 3.

List of antibodies used in this study.
