# Supplementary material for: Downregulation of chemokine receptor 9 facilitates CD4+CD8αα+ intraepithelial lymphocyte development
Source: Nat Commun. 2023 Aug 24;14:5152. doi: 10.1038/s41467-023-40950-2 (PMC10449822; doi:10.1038/s41467-023-40950-2)
Supplement: Supplementary file 7 — Reporting Summary [file 41467_2023_40950_MOESM7_ESM.pdf]

nature portfolio

Corresponding author(s): Tomohia Sujino

Last updated by author(s): 26/7/23

Reporting Summary

Nature Portfolio wishes to improve the reproducibility of the work that we publish. This form provides structure for consistency and transparency in reporting. For further information on Nature Portfolio policies, see our Editorial Policies and the Editorial Policy Checklist.

Statistics

For all statistical analyses, confirm that the following items are present in the figure legend, table legend, main text, or Methods section.

n/a	Confirmed	
☐	☒	The exact sample size (n) for each experimental group/condition, given as a discrete number and unit of measurement
☐	☒	A statement on whether measurements were taken from distinct samples or whether the same sample was measured repeatedly
☐	☒	The statistical test(s) used AND whether they are one- or two-sided<br>*Only common tests should be described solely by name; describe more complex techniques in the Methods section.*
☐	☒	A description of all covariates tested
☐	☒	A description of any assumptions or corrections, such as tests of normality and adjustment for multiple comparisons
☐	☒	A full description of the statistical parameters including central tendency (e.g. means) or other basic estimates (e.g. regression coefficient) AND variation (e.g. standard deviation) or associated estimates of uncertainty (e.g. confidence intervals)
☐	☒	For null hypothesis testing, the test statistic (e.g. F, t, r) with confidence intervals, effect sizes, degrees of freedom and P value noted<br>*Give P values as exact values whenever suitable.*
☒	☐	For Bayesian analysis, information on the choice of priors and Markov chain Monte Carlo settings
☒	☐	For hierarchical and complex designs, identification of the appropriate level for tests and full reporting of outcomes
☒	☐	Estimates of effect sizes (e.g. Cohen's d, Pearson's r), indicating how they were calculated

Our web collection on statistics for biologists contains articles on many of the points above.

Software and code

Policy information about availability of computer code

Data collection	BD FACS Diva version 8.0.1 for flowcytometry.
Data analysis	Flow cytometry data was analyzed by FlowJo (version 10). GraphPad Prism 6 was used for statistical analysis. Bowtie2 (version 2.1.0) and Homer (version 4.10) was used for ChIP-seq. QIIME software package v1.9.1, ea-utils software v1.1.2 for 16S rRNA analysis. The following software was used for scRNA-seq/TCR-seq analysis:Cell Ranger (v.7.0.1), Seurat (v.4.0.6), scanpy package (v.1.9.1), divo(R) (v.1.0.1), seaborn heatmap (v.0.11.2), skbio. diversity. alpha (v.0.4.2), Monocle 3 v3.0, and seaborn violinplot (v.0.11.2).

For manuscripts utilizing custom algorithms or software that are central to the research but not yet described in published literature, software must be made available to editors and reviewers. We strongly encourage code deposition in a community repository (e.g. GitHub). See the Nature Portfolio guidelines for submitting code & software for further information.

Data

Policy information about availability of data

All manuscripts must include a data availability statement. This statement should provide the following information, where applicable:

- Accession codes, unique identifiers, or web links for publicly available datasets
- A description of any restrictions on data availability
- For clinical datasets or third party data, please ensure that the statement adheres to our policy

The data for ChIP-seq and scRNA-seq have been deposited in DNA Data Bank of Japan. Accession numbers are DRA014018 (https://ddbj.nig.ac.jp/resource/sra-submission/DRA014018) for ChIP-seq, and DRA015828 (https://ddbj.nig.ac.jp/resource/sra-submission/DRA015828) and DRA014019 (https://ddbj.nig.ac.jp/resource/sra-submission/DRA014019) for scRNA-seq. All data available in a publicly accessible repository. ChIP-seq based on mm9 mouse genome reference. scRNA-seq based on mm10 mouse genome reference.

Field-specific reporting

Please select the one below that is the best fit for your research. If you are not sure, read the appropriate sections before making your selection.

- [x] Life sciences
- [ ] Behavioural & social sciences
- [ ] Ecological, evolutionary & environmental sciences

For a reference copy of the document with all sections, see nature.com/documents/nr-reporting-summary-flat.pdf

Life sciences study design

All studies must disclose on these points even when the disclosure is negative.

Sample size	Sample size for each experiment is indicated in the figures and figure legends for each experiment. No statistical methods were used to predetermine sample size.
Data exclusions	No data was excluded from the analysis.
Replication	Each experiment was replicated more than two times with reproducible results.
Randomization	No systemic randomization was used for animal studies since methods including subjective measures were not used in this study. Mice were matched based on gender (male), ages, litters, and cages to remove experimental bias.
Blinding	The data collection and analysis were not blinded. Blinding was not possible as the investigators were also conducting the experiments and had to be aware of groups of analysis.

Reporting for specific materials, systems and methods

We require information from authors about some types of materials, experimental systems and methods used in many studies. Here, indicate whether each material, system or method listed is relevant to your study. If you are not sure if a list item applies to your research, read the appropriate section before selecting a response.

Materials & experimental systems

n/a	Involved in the study	
☐	☒	Antibodies
☒	☐	Eukaryotic cell lines
☒	☐	Palaeontology and archaeology
☐	☒	Animals and other organisms
☒	☐	Human research participants
☒	☐	Clinical data
☒	☐	Dual use research of concern

Methods

n/a	Involved in the study	
☐	☒	ChIP-seq
☐	☒	Flow cytometry
☒	☐	MRI-based neuroimaging

Antibodies

Antibodies used	For in vitro T cell culture, the following antibodies were used: 1ug/ml anti-CD3e antibody (Biolegend, clone 145-2C11, cat 100359) with 1ug/ml soluble anti-CD28 antibody (Biolegend, clone 37.51, cat 102116).<br>For flowcytometry analysis, antibodies were purchased from BD Biosciences, Biolegend, and Thermo Fisher Scientific: anti-mouse CD4 (Biolegend, APC/BV421, clone RM4-5, cat 100516/100544, 1/200), anti-mouse CD8a (BD Biosciences, BV500/PE-Cy7, clone 53-6.7, cat 560776/552877, 1/200), anti-mouse CD8b (Thermo Fisher Scientific, FITC/APC, clone eBioH35-17.2, cat 11-0083-85/17-0083-81, 1/200), anti-mouse/human CD44 (Biolegend, APC, clone IM7, cat 103012, 1/200), anti-mouse CD45 (Biolegend, BV510, clone 30-F11, cat 103138, 1/200), anti-mouse CD45.1 (BD Biosciences, FITC, clone A20, cat 553775, 1/200), anti-mouse CD45.2 (Biolegend, BV510/PE-Cy7, clone 104, cat 109838/109830, 1/200), anti-mouse CD62L (Biolegend, PerCP-Cy5.5, clone MEL-14, cat 104432, 1/200), anti-mouse TCRb (Biolegend, APC-Cy7/PE-Cy7, clone H57-597, cat 109220/109222, 1/200), anti-mouse TCRgd (Biolegend, PerCP-Cy5.5, clone GL3, cat 118117, 1/200), anti-mouse a4b7 (BD Biosciences, PE, clone DATK32, cat 553811, 1/100), anti-mouse CCR9 (BD Biosciences, BV421, clone CW-1.2, cat 565412, 1/100), anti-mouse Foxp3 (Thermo Fisher Scientific, PE/PerCP-Cy5.5, clone FJK-16s, cat 12-5773-82/45-5773-82, 1/200), anti-mouse Ki67 (Biolegend, PE, clone 16A8, ct 652403, 1/100), anti-mouse IL17A (Thermo Fisher Scientific, PE, clone eBio17B7, cat 12-7177-81, 1/100), anti-mouse IFNg (BD Biosciences, PE-Cy7, clone XMG1.2, cat 557649, 1/100), and fixable viability dye (FVD) (Thermo Fisher Scientific, APC-Cy7, cat 65-0865-14, 1/1000).<br>For ChIP-seq analysis, rabbit anti-mouse Cbfb2 antibody was used (produced by Taniuchi lab).
Validation	Anti-CD3e (Biolegend, clone 145-2C11, cat 100359) https://www.biolegend.com/ja-jp/products/ultra-leaf-purified-anti-mouse-cd3epsilon-antibody-7722<br><br>Anti-CD28 (Biolegend, clone 37.51, cat 102116) https://www.biolegend.com/ja-jp/products/ultra-leaf-purified-anti-mouse-cd28-antibody-7733

Anti-mouse CD4 (Biolegend, APC, clone RM4-5, 100516) https://www.biolegend.com/ja-jp/products/apc-anti-mouse-cd4-antibody-477

Anti-mouse CD4 (Biolegend, BV421, clone RM4-5, 100544) https://www.biolegend.com/ja-jp/products/brilliant-violet-421-anti-mouse-cd4-antibody-7349

Anti-mouse CD8a (BD Biosciences, V500, clone 53-6.7, 560776) https://www.bdbiosciences.com/ja-jp/products/reagents/flow-cytometry-reagents/research-reagents/single-color-antibodies-ruo/v500-rat-anti-mouse-cd8a.560776

Anti-mouse CD8a (BD Biosciences, PE-Cy7, clone 53-6.7, 552877) https://www.bdbiosciences.com/ja-jp/products/reagents/flow-cytometry-reagents/research-reagents/single-color-antibodies-ruo/pe-cy-7-rat-anti-mouse-cd8a.552877

Anti-mouse CD8b (Thermo Fisher Scientific, FITC, clone eBioH35-17.2, 11-0083-85) https://www.thermofisher.com/antibody/product/CD8b-Antibody-clone-eBioH35-17-2-H35-17-2-Monoclonal/11-0083-85

Anti-mouse CD8b (Thermo Fisher Scientific, APC, clone eBioH35-17.2, 17-0083-81) https://www.thermofisher.com/antibody/product/CD8b-Antibody-clone-eBioH35-17-2-H35-17-2-Monoclonal/17-0083-81

Anti-mouse/human CD44 (Biolegend, APC, clone IM7, 103012) https://www.biolegend.com/ja-jp/products/apc-anti-mouse-human-cd44-antibody-312

Anti-mouse CD45 (Biolegend, BV510, clone 30-F11, 103138) https://www.biolegend.com/ja-jp/products/brilliant-violet-510-anti-mouse-cd45-antibody-7995

Anti-mouse CD45.1 (BD Biosciences, FITC, cone A20, 553775) https://www.bdbiosciences.com/ja-jp/products/reagents/flow-cytometry-reagents/research-reagents/single-color-antibodies-ruo/fitc-mouse-anti-mouse-cd45-1.553775

Anti-mouse CD45.2 (Biolegend, BV510, clone 104, 109838) https://www.biolegend.com/ja-jp/products/brilliant-violet-510-anti-mouse-cd45-2-antibody-7998

Anti-mouse CD45.2 (Biolegend, PE-Cy7, clone 104, 109830) https://www.biolegend.com/ja-jp/products/pe-cyanine7-anti-mouse-cd45-2-antibody-4918

Anti-mouse CD62L (Biolegend, PerCP-Cy5.5, clone MEL-14, 104432) https://www.biolegend.com/ja-jp/products/percp-cyanine5-5-anti-mouse-cd62l-antibody-4272

Anti-mouse TCRb (Biolegend, APC-Cy7, clone H57-597, 109220) https://www.biolegend.com/ja-jp/products/apc-cyanine7-anti-mouse-tcr-beta-chain-antibody-4137

Anti-mouse TCRb (Biolegend, PE-Cy7, clone H57-597, 109222) https://www.biolegend.com/ja-jp/products/pe-cyanine7-anti-mouse-tcr-beta-chain-antibody-4144

Anti-mouse TCRgd (Biolegend, PerVP-Cy5.5, clone GL3, 118117) https://www.biolegend.com/ja-jp/products/percp-cyanine5-5-anti-mouse-tcr-gamma-delta-antibody-6702

Anti-mouse a4b7 (BD Biosciences, PE, clone DATK32, 553811) https://www.bdbiosciences.com/ja-jp/products/reagents/flow-cytometry-reagents/research-reagents/single-color-antibodies-ruo/pe-rat-anti-mouse-lpam-1.553811

Anti-mouse CCR9 (BD Biosciences, BV421, clone CW-1.2,565412) https://www.bdbiosciences.com/ja-jp/products/reagents/flow-cytometry-reagents/research-reagents/single-color-antibodies-ruo/bv421-mouse-anti-mouse-cd199-ccr9.565412

Anti-mouse Foxp3 (Thermo Fisher Scientific, PE, clone FJK-16s, 12-5773-82) https://www.thermofisher.com/antibody/product/FOXP3-Antibody-clone-FJK-16s-Monoclonal/12-5773-82

Anti-mouse Foxp3 (Thermo Fisher Scientific, PerCP-Cy5.5, clone FJK-16s, 45-5773-82) https://www.thermofisher.com/antibody/product/FOXP3-Antibody-clone-FJK-16s-Monoclonal/45-5773-82

Anti-mouse Ki67 (Biolegend, PE, clone 16A8, 652403) https://www.biolegend.com/ja-jp/products/pe-anti-mouse-ki-67-antibody-8134

Anti-mouse IL17A (Thermo Fisher Scientific, PE, clone eBio17B7, 12-7177-81) https://www.thermofisher.com/antibody/product/IL-17A-Antibody-clone-eBio17B7-Monoclonal/12-7177-81

Anti-mouse IFNg (BD Biosciences, PE-Cy7, clone XMG1.2, 557649) https://www.bdbiosciences.com/ja-jp/products/reagents/flow-cytometry-reagents/research-reagents/single-color-antibodies-ruo/pe-cy-7-rat-anti-mouse-ifn.557649

Anti-mouse CD16/32 (CD16/32, BD Biosciences, 553141) https://www.bdbiosciences.com/ja-jp/products/reagents/flow-cytometry-reagents/research-reagents/single-color-antibodies-ruo/purified-rat-anti-mouse-cd16-cd32-mouse-bd-fc-block.553141

Fixable Viability dye (Thermo Fisher Scientific, 65-0865-14) https://www.thermofisher.com/order/catalog/product/65-0865-14?SID=srch-srp-65-0865-14

Rabbit anti mouse Cbfb2 antibody was produced by Taniuchi lab (Naoe et al. JEM 2007)

Animals and other organisms

Policy information about studies involving animals; ARRIVE guidelines recommended for reporting animal research

Laboratory animals	C57BL/6J mice were purchased from CLEA Japan, INC. (Tokyo, Japan). Ccr9−/−, Ly5.1, Rag2−/−, and germ-free mice were purchased from Sankyo Labo Service Corporation, INC (Tokyo, Japan). ThpokGFP:Runx3tdTomato reporter, Cbfβ2m/2m, Rosa26lsl-cbfb2-GFP, Cd4-Cre, Cbfb1tdTomato, Cbfb2Venus reporter, and Rosa26lsl-Ccr9-GFP mice were provided by Dr Ichiro Taniuchi (Riken Center for Integrative Medical Sciences, Yokohama, Japan). Ccl25+/+ and Ccl25-/- mice were provided by Dr Kazutoshi Sayama (Shizuoka University, Shizuoka, Japan). Mice, except for germ-free mice, were maintained under specific pathogen–free (SPF) conditions with a 12h light/dark cycle, at a temperature of 22-25 °C and a relative humidity of 45-55 % in the Animal Care Facility of Keio University School of Medicine. Germ-free mice were bred and maintained in vinyl isolators. Male mice, and aged 7 to 17 weeks were used.
Wild animals	This study did not involve wild animals.
Field-collected samples	This study did not involve samples collected from the field.
Ethics oversight	Animal Ethics Committee of Keio University approved all animal studies.

Note that full information on the approval of the study protocol must also be provided in the manuscript.

ChIP-seq

Data deposition

☒ Confirm that both raw and final processed data have been deposited in a public database such as GEO.

☒ Confirm that you have deposited or provided access to graph files (e.g. BED files) for the called peaks.

Data access links<br>*May remain private before publication.*	https://ddbj.nig.ac.jp/resource/sra-submission/DRA014018
Files in database submission	DRS259681<br>DRS259682<br>DRS259683
Genome browser session<br>(e.g. UCSC)	No longer applicable.

Methodology

Replicates	ChIP-seq experiments were performed twice and merged data were deposited.
Sequencing depth	More than 30,000,000 reads are mapped in each ChIP-seq sample.
Antibodies	Rabbit anti mouse CBFb antibody was produced by our lab (Naoe et al., J Exp Med 2007).
Peak calling parameters	ChIP peaks were identified using Homer (version 4.10) with the default parameters.
Data quality	Peaks were called using Homer (version 4.10) with the default parameters.
Software	Sequencer signals were converted into nucleotide sequences using Illumina offline basecaller (OLB) version 1.9.4.<br>Sequences were mapped on mouse genome using bowtie2 (version 2.1.0).<br>Peak calling was done by Homer (version 4.10).

Flow Cytometry

Plots

Confirm that:

- [x] The axis labels state the marker and fluorochrome used (e.g. CD4-FITC).
- [x] The axis scales are clearly visible. Include numbers along axes only for bottom left plot of group (a 'group' is an analysis of identical markers).
- [x] All plots are contour plots with outliers or pseudocolor plots.
- [x] A numerical value for number of cells or percentage (with statistics) is provided.

Methodology

Sample preparation	Small intestine was cutted into small pieces and washed with calcium- and magnesium-free Hank's balanced salt solution (HBSS) (Nacalai Tesque). After washing, the small intestine was incubated with HBSS containing 1 mM dithiothreitol (Thermo Fisher Scientific K.K) and 5 mM EDTA (Thermo Fisher Scientific K.K) for 30 min at 37°C to remove the epithelial layer. The mucosal pieces were then washed and dissolved in solution by incubation with HBSS containing 1.5% fetal bovine serum (Thermo Fisher Scientific K.K), 1.0 mg/mL collagenase (Wako), and 0.1 mg/mL DNase (Sigma-Aldrich) for 30 min at 37 °C. The dissolved solution was centrifuged and the resulting pellet was resuspended in 40% Percoll (GE Healthcare) and overlaid on 75% Percoll. Percoll gradient separation was performed by centrifugation at 840 ×g and 20 °C for 20 minutes. LP mononuclear cells were collected at the interphase between 40% and 75% Percoll layers. For IELs, the supernatant containing the epithelial layers was collected and centrifuged. The resulting pellet was resuspended, separated, and collected following the procedures used for mucosal tissue.<br>MLN and spleen tissues were homogenized manually in HBSS. The lysates were filtered through a cell strainer. MLN cells were centrifuged and collected. Splenic cells were hemolyzed with 0.84% (v/w) ammonium chloride (Nacalai Tesque), washed with HBSS, and collected for analysis.
Instrument	Isolated cells were stained with fluorochrome-conjugated antibody.<br>For cell sorting: FACSAria II (Becton Dickinson).<br>For analysis: FACSCanto II (Becton Dickinson)
Software	FACSDiva for data collection and FlowJo (v10) for analysis.
Cell population abundance	Populations were validated for purity by a post-sort analysis by FACS.
Gating strategy	FSC/SSC were used for lymphocytes, FSC-H/FSC-W and SSC-H/SSC-W for single cells and FVD for viable cells.

- [x] Tick this box to confirm that a figure exemplifying the gating strategy is provided in the Supplementary Information.
